# Time-Course Comparative Transcriptomics Between Isolates of *Cercospora zeae-maydis* and *Cercospora zeina* During Maize Infection

**DOI:** 10.3390/jof12090650

**Published:** 2026-09-01

**Authors:** Zhaoreng Li, Ranran Zhang, Xiaoshan Tan, Lifang Guo, Jili Deng, Shengjie Gong, Luchen Li, Chengcheng Li, Ying Hu, Zhao Peng

**Affiliations:** 1 College of Plant Protection, Jilin Agricultural University, Changchun 130118, China; lizhaoreng@gmail.com (Z.L.); 18247590490@163.com (R.Z.); tanxiaoshan1964@163.com (X.T.); lfgou689@163.com (L.G.); jldeng37@163.com (J.D.); shengjiegong2023@163.com (S.G.); luchenli2024@163.com (L.L.); 13730529013@163.com (C.L.); 2 Engineering Research Center of Natural Enemies, Institute of Biological Control, Jilin Agricultural University, Changchun 130118, China; huying@jlau.edu.cn

**Keywords:** *Cercospora zeae-maydis*, *Cercospora zeina*, maize gray leaf spot, Jilin isolates, pathogen-focused RNA-seq, candidate effectors, cell wall-degrading enzymes, cercosporin

## Abstract

Gray leaf spot (GLS) is a major foliar disease of maize caused mainly by *Cercospora zeae-maydis* and *Cercospora zeina*. Although both species infect maize, the comparative transcriptional programs between isolates of different species during maize colonization remain largely unknown. We conducted time-course pathogen-focused RNA-seq of one Jilin isolate assigned to each species during colonization of maize B73 under a controlled inoculation system. RNA-seq reads were mapped to the public *C. zeae-maydis* SCOH1-5 and *C. zeina* CMW25467 v3 reference genomes for transcript quantification and putative functional annotation. The *C. zeae-maydis* isolate developed visible symptoms earlier and showed greater pathogen-derived transcript representation at 6 dpi. The *C. zeae-maydis* isolate expressed slightly more reference-annotated cell wall-degrading enzyme (CWDE)-associated loci than the *C. zeina* isolate, whereas the numbers of expressed candidate effector-associated loci were similar. The *C. zeae-maydis* isolate also showed earlier or stronger host-associated induction of several CWDE- and candidate effector-encoding genes. Both isolates showed post-inoculation induction of candidate transcripts related to lipid metabolism, protease-related functions, and cercosporin biosynthesis, while a subset differed between isolates in the timing or magnitude of expression. RT-qPCR broadly supported the temporal trends of selected candidates. Together, these results define shared and contrasting transcriptional programs of two isolates of different *Cercospora* species during maize colonization.

## 1. Introduction

Gray leaf spot (GLS), caused by fungi in the genus *Cercospora*, has emerged as a globally significant foliar disease of maize (*Zea mays* L.). The prevalence of GLS is exacerbated by the increased adoption of reduced tillage systems, use of susceptible maize hybrids, high planting densities, and climate warming, which collectively foster humid, prolonged leaf-wetness microenvironments conducive to disease [1]. First reported in the United States, GLS has since spread to Africa, Brazil, and China [2,3]. It is now affecting both northern and southern maize-growing regions and leading to significant reductions in yield and grain quality in China [4].

The GLS disease complex of maize is primarily caused by two closely related but distinct species, *Cercospora zeae-maydis* and *Cercospora zeina* [5]. While the two pathogens belong to the same *Cercospora* species complex and are phylogenetically close, previous studies have reported differences in geographic distribution, climatic adaptation, and virulence among isolates assigned to these species [6]. The initial outbreaks in the southern maize belt of North America were attributed to *C. zeae-maydis* [1]. However, subsequent phylogenetic studies identified *C. zeina* as the predominant species in subtropical regions, including southern Africa and parts of China, indicating divergence in climatic adaptation and regional niche preference [3,4]. Genomic comparisons have provided insights into pathogenicity-associated gene repertoire variation in *C. zeae-maydis* and *C. zeina* reference genomes and isolates [7]. However, side-by-side pathogen-focused transcriptomic comparisons of *C. zeae-maydis* and *C. zeina* under the same maize inoculation and analytical framework remain limited.

The plant cell wall is a primary physical barrier, which is composed of a network of cellulose, hemicellulose, pectin and other structural components [8,9]. To breach this cell wall barrier, fungal pathogens secrete an arsenal of Carbohydrate-Active Enzymes (CAZymes), including glycoside hydrolases (GHs), polysaccharide lyases (PLs), and carbohydrate esterases (CEs), which depolymerize wall polysaccharides to facilitate host invasion and nutrient acquisition [10,11]. The secretion of these enzymes is a highly regulated process, wherein the pathogen produces a specific set of CAZymes adapted to the host’s cell wall composition [10,12]. However, side-by-side transcriptomic profiling of CAZyme gene expression in representative *C. zeae-maydis* and *C. zeina* isolates under the same inoculation framework remains limited, leaving a gap in our understanding of conserved and variable cell wall degradation-related responses.

The infection cycle of *Cercospora* spp. is characterized by a hemibiotrophic lifestyle. Following stomatal penetration or direct epidermal invasion, the pathogen enters an extended latent period with limited symptom development [13]. During the latent phase, pathogens typically secrete an arsenal of effector proteins to suppress plant immunity. The first layer of defense, Pattern-Triggered Immunity (PTI), is activated when plant pattern-recognition receptors (PRRs) detect pathogen-associated molecular patterns (PAMPs) like chitin [14]. Fungal effectors, such as the chitin-binding Avr4 or the chitin-sequestering Ecp6 of *Cladosporium fulvum*, can shield these PAMPs to evade detection [15,16]. Extensive research has uncovered the multifaceted roles of fungal effectors in suppressing plant immune responses [14]. In addition to inhibiting plant defenses, these effectors are also reported to manipulate plant physiology, facilitating fungal colonization and providing nutrients to support the invading pathogens [17,18]. Despite extensive research on fungal effectors, side-by-side transcriptomic studies examining the temporal expression and isolate-level variation in effector repertoires between representative *C. zeae-maydis* and *C. zeina* isolates during maize colonization remain limited. Such analyses can identify candidate virulence-associated mechanisms for future validation across broader isolate collections.

The transition from latent biotrophy to destructive necrotrophy is a critical phase in disease development, and is critically dependent on light, which activates the biosynthesis of the photoactivated perylenequinone toxin, cercosporin [13,19]. Its production is governed by the cercosporin toxin biosynthesis (CTB) gene cluster, wherein the polyketide synthase CTB1 serves as the backbone enzyme, with CTB2 and CTB3 involved in methyl transfer and redox modifications [20,21,22]. Further studies revealed the involvement of other *CTB* genes in cercosporin biosynthesis [23,24]. Cercosporin generates a burst of reactive oxygen species (ROS) in host tissues, inducing programmed cell death and the formation of the characteristic gray necrotic lesions [13,19]. The timing and regulatory mechanisms of this latent-to-necrotrophic shift may vary across *Cercospora* species, isolates, and host–pathogen interactions, suggesting potential divergence in pathogenicity-related processes [25]. However, matched comparisons of CTB gene expression between representative *C. zeae-maydis* and *C. zeina* isolates under the same maize inoculation and sampling framework remain limited.

To address these knowledge gaps, we performed time-course pathogen-focused transcriptome profiling of one Jilin isolate assigned to *C. zeae-maydis* and one Jilin isolate assigned to *C. zeina* under a common controlled maize leaf inoculation framework. The public *C. zeae-maydis* SCOH1-5 and *C. zeina* CMW25467 v3 reference genomes supported read mapping, transcript quantification, and putative functional annotation, while reference-based orthogroup analysis and analysis of genes associated with cercosporin biosynthesis established the annotation context for the expressed transcripts. By integrating symptom development, pathogen-derived transcript representation, temporal RNA-seq profiles, and RT-qPCR validation, we examined the shared colonization-associated transcriptional program of the two isolates and identified candidate transcript groups that distinguished their temporal responses during maize leaf colonization.

## 2. Materials and Methods

### 2.1. Fungal Strains, Identification, and Inoculum Preparation

One isolate assigned to *C. zeae-maydis* (hereafter CM) and one isolate assigned to *C. zeina* (hereafter CZ) were obtained from maize leaves showing GLS symptoms in Jilin Province, China. These two isolates were selected for a controlled, side-by-side comparison of maize colonization and pathogen-focused transcriptional responses. Initial fungal identity was assessed by PCR amplification and Sanger sequencing of the internal transcribed spacer (ITS) region. Genomic DNA was extracted from fresh fungal mycelium using a CTAB-based method [26]. The ITS region was amplified with primers ITS1 (5′-TCCGTAGGTGAACCTGCGG-3′) and ITS4 (5′-TCCTCCGCTTATTGATATGC-3′). PCR products were purified and bidirectionally sequenced by Sanger sequencing. The ITS sequences of the CM and CZ Jilin isolates were deposited in GenBank under accession numbers OR945717 and PV133732, respectively [27]. Final taxonomic assignment was further supported by multilocus phylogenetic analysis as described below. Genome sequencing of these two Jilin isolates was not performed in this study; therefore, subsequent RNA-seq quantification and functional interpretation relied on mapping reads to publicly available reference genomes of *C. zeae-maydis* SCOH1-5 and *C. zeina* CMW25467 v3. For phenotypic documentation of in vitro growth and sporulation culture morphology, the two representative isolates were cultured on potato dextrose agar (PDA) medium and maize leaf powder sporulation medium for 8 days at 25 °C. The maize leaf powder sporulation medium was prepared based on a previously described maize leaf powder–CaCO_3_ agar formulation for *Cercospora zeae-maydis*, with minor modifications [28]. The medium contained 15 g maize leaf powder, 1.5 g CaCO_3_, 15 g agar, and 1000 mL distilled water. In the present study, the maize leaf powder was prepared from healthy leaves collected from uninoculated B73 plants, oven-dried until fully dry, ground into a fine powder, and stored dry at room temperature until use. B73 was the same maize genotype used in the inoculation assay and was treated as a susceptible host genotype under the conditions of this study. Mycelial biomass was evenly spread over the agar surface to promote conidiation. Representative colony and sporulation culture images were recorded and are shown in Figure S1.

For inoculum production, the two isolates were cultured on maize leaf powder sporulation medium at 25 °C to promote conidiation. Spores were harvested by rinsing sporulating cultures with sterile distilled water and filtering the suspension through sterile gauze to remove mycelial fragments. Spore concentrations were determined using a hemocytometer and adjusted to 1.6 × 10^5^ spores/mL for *C. zeae-maydis* and 6.4 × 10^5^ spores/mL for *C. zeina*, as the latter produced few or no visible lesions at lower concentrations under our experimental conditions. These concentrations were selected to obtain reproducible infection and sufficient host-associated fungal signal for subsequent analyses, rather than to directly compare aggressiveness between the two isolates or species on an equal-inoculum basis.

### 2.2. Multilocus Phylogenetic Analysis for Isolate Identification

To confirm the taxonomic identity of the two *Cercospora* isolates used in this study, a multilocus phylogenetic analysis was performed using five loci: the internal transcribed spacer region (ITS), translation elongation factor 1-alpha (TEF1), actin (ACT), calmodulin (CAL), and histone H3 (HIS3). Reference marker sequences representing *C. zeae-maydis*, *C. zeina*, and closely related *Cercospora* and *Pseudocercospora* species were retrieved from the NCBI nucleotide database using Entrez Direct v22.4 [29]. For reference strains, published NCBI marker sequences were preferentially used when strain-specific accessions were available. Genome-derived marker sequences extracted from publicly available genome assemblies were retained only for loci or strains lacking corresponding strain-specific published marker accessions. UNVERIFIED sequences and short sequences were excluded, and the retained sequences were manually checked. The accession numbers and sources of all sequences used in the phylogenetic analysis are listed in Table S1.

For the two Jilin isolates sequenced in this study, protein-coding marker sequences were identified from Trinity v2.11.0 [30] de novo transcriptome assemblies by BLASTN searches implemented in NCBI BLAST+ v2.16.0 [31] against the curated reference marker set. Candidate marker regions were extracted, oriented according to the BLAST hit direction, and validated by reciprocal BLASTN searches against the reference marker database. The ITS sequences of the two isolates were represented by GenBank accessions OR945717 and PV133732, respectively.

Sequences for each locus were aligned separately using MAFFT v7 [32] with the --auto option and trimmed using trimAl v1.5.rev1 [33] with the -automated1 option. The trimmed alignments were concatenated using AMAS [34]. Maximum-likelihood phylogenetic inference was performed using IQ-TREE v3.0.1 [35] with ModelFinder [36], partition merging (-m MFP+ MERGE), 1000 ultrafast bootstrap replicates. The resulting tree was rooted with *Pseudocercospora griseola*. Pairwise p-distances were calculated from the same concatenated trimmed alignment to compare the two Jilin isolates with the corresponding *C. zeae-maydis* and *C. zeina* reference sequence sets.

### 2.3. Controlled Plant Inoculation and Sample Collection

Maize inbred line B73 seeds were kindly provided by Dr. Jun Zheng from the Chinese Academy of Agricultural Sciences. Samples were collected from both host-associated inoculated leaves and in vitro fungal culture conditions. For the in vitro fungal RNA-seq samples, the two Jilin isolates were grown on maize leaf powder sporulation medium under the same temperature condition used for inoculum production. Briefly, actively growing fungal biomass was transferred onto maize leaf powder sporulation medium and incubated at 25 °C for 8 days. Mycelial biomass, together with sporulation-associated fungal material from the colony surface, was collected using a sterile scalpel, immediately frozen in liquid nitrogen, and stored at −80 °C until RNA extraction. Three independently processed culture samples were prepared for each isolate. These in vitro samples provided a non-host fungal reference for visualization and exploratory comparison, whereas primary DESeq2 v1.48.1 testing used the corresponding 0 dpi inoculation samples as the baseline.

B73 seedlings at the three-leaf stage were inoculated on the second leaves using a needleless syringe-assisted inoculation method. Spore suspensions of each pathogen were gently applied to the abaxial leaf surface with light pressure to promote localized and reproducible contact between the inoculum and leaf tissue. This controlled inoculation system was used to generate synchronized host-associated fungal samples for comparative transcriptome profiling and was not intended to fully reproduce natural stomatal penetration under field conditions. Inoculated plants were maintained in a growth chamber under a 14-h light/10-h dark photoperiod at 28 °C and 90% relative humidity. Leaf tissues were harvested at defined time points to construct a temporal host-associated fungal transcriptional profile. The 0, 1, 3, and 6 dpi time points were selected to capture the progression from the inoculation baseline and early host contact to the onset of visible lesion development under the controlled inoculation system. The 0 dpi samples were collected 30 min after inoculation and used as the inoculation baseline for comparisons with samples collected at 1, 3, and 6 dpi. The 6 dpi time point was chosen as the final RNA-seq sampling point because visible lesions were observed in CZ-inoculated leaves by this time, whereas CM-inoculated leaves already showed more advanced symptom development.

For each pathogen and post-inoculation time point, three independently processed pooled samples were prepared. Each pooled sample comprised leaf tissue from three or four inoculated leaves and was processed separately for RNA extraction, library preparation, and sequencing. The three independently processed pooled samples were used for visualization and exploratory statistical summaries of pathogen-derived read mapping rates and gene expression patterns. In addition to the samples collected for RNA-seq, inoculated leaves were maintained for extended symptom observation to document symptom reproduction by the inoculated isolates. Representative inoculated leaves were photographed at 15 dpi for CM and 20 dpi for CZ to document the late-stage development of rectangular gray leaf spot lesions delimited by leaf veins (Figure S1). In total, this experimental design yielded 30 RNA-seq samples for pathogen-focused comparative transcriptomic analysis.

### 2.4. RNA Sequencing and Data Processing

Total RNA was extracted from harvested inoculated leaf tissues and from the in vitro fungal culture samples described above using the Ultrapure RNA Kit (CWBio, Taizhou, China) according to the manufacturer’s instructions. RNA quality and integrity were assessed using a NanoDrop 2000 spectrophotometer (Thermo Fisher Scientific, Waltham, MA, USA) and an Agilent 2100 Bioanalyzer (Agilent Technologies, Santa Clara, CA, USA), with all samples having an RNA Integrity Number (RIN) ≥ 7.0. Sequencing libraries were prepared by enriching poly(A)+ mRNA with Oligo(dT) magnetic beads and constructing non-strand-specific, paired-end libraries. The libraries were then sequenced on an Illumina NovaSeq 6000 platform (Biomarker, Beijing, China) to generate 150 bp paired-end reads.

Raw Illumina reads were quality-filtered using Trimmomatic v0.39 to remove adapter sequences and low-quality bases [37]. To distinguish pathogen-derived transcripts from host transcripts, host–pathogen interaction reads were aligned to the corresponding maize–pathogen combined reference genomes using HISAT2 v2.2.1 [38]. The maize reference genome Zm-B73-REFERENCE-NAM-5.0, the *C. zeae-maydis* strain SCOH1-5 genome assembly Cerzm1 (GCA_010093985.1), and the *C. zeina* strain CMW25467 genome assembly ASM284461v3 (GCA_002844615.3) were used for reference construction. Before concatenation with the maize reference genome, pathogen genome sequence identifiers were prefixed with pathogen-specific tags to avoid sequence name conflicts. In vitro fungal culture samples were aligned to the corresponding pathogen genomes, whereas host–pathogen interaction samples were aligned to the corresponding maize–pathogen combined references. Alignments were converted to sorted and indexed BAM files using SAMtools v1.15 [39]. For combined-reference alignments, BAM files were further separated into maize-derived and pathogen-derived BAM files according to reference sequence origin.

Expression quantification was performed using featureCounts v2.0.6 [40]. Pathogen-derived reads from the two Jilin isolates were quantified against the corresponding public reference gene annotations. Accordingly, pathogen gene identifiers reported in the RNA-seq analysis denote the *C. zeae-maydis* SCOH1-5 or *C. zeina* CMW25467 v3 reference gene models supported by Jilin isolate-derived reads, and the reported functional identities represent putative reference-guided assignments to the corresponding expressed transcripts. For maize samples, exon-overlapping reads were summarized at the gene_id level based on the maize annotation file, whereas for pathogen samples, exon-overlapping reads were summarized at the transcript_id level based on the corresponding pathogen annotation files. Differential expression analysis was performed separately for *C. zeae-maydis* and *C. zeina* using DESeq2 [41]. For each isolate, differential expression was assessed by comparing samples collected at 1, 3, and 6 dpi with the corresponding 0 dpi samples collected 30 min after inoculation. Genes showing low expression at 0 dpi but meeting the exploratory DESeq2 criteria at later time points were designated candidate post-inoculation-induced transcripts.

To reduce the influence of extremely low-count genes on differential expression testing, low-expression genes were filtered prior to DESeq2 analysis. For DEG calling, genes were retained only when they had at least 10 raw counts in at least three samples within the corresponding isolate-specific dataset. DESeq2 analysis was performed using raw read counts, and transcripts with FDR < 0.05 and |log2 FoldChange| > 1 were retained as exploratory differentially expressed transcript candidates. Normalized FPKM values were used only for visualization of expression trends in heatmaps. Statistical inference for differential expression was based on raw read counts analyzed with DESeq2. For selected analyses of host-associated expression patterns, FPKM profiles from the in vitro fungal cultures were examined descriptively together with the 0 dpi and post-inoculation samples. Candidates showing low FPKM in vitro and at 0 dpi but increased FPKM at one or more post-inoculation time points were selected for temporal visualization. DESeq2 testing remained restricted to comparisons of 1, 3, and 6 dpi with the corresponding 0 dpi baseline within each isolate.

### 2.5. Reference-Based Orthogroup Classification for Functional Annotation

All reference genome sequences and corresponding GFF3 annotation files were downloaded from the NCBI website. The *C. zeae-maydis* strain SCOH1-5 genome assembly Cerzm1 (GCA_010093985.1) and the *C. zeina* strain CMW25467 genome assembly ASM284461v3 (GCA_002844615.3) [42] were used as the primary references because both provided genome sequences and corresponding gene annotation files required for reference-guided expression quantification, functional annotation, and orthogroup-based contextual interpretation of mapped transcripts. Although a higher-quality long-read *C. zeae-maydis* genome assembly has been reported, a corresponding public GFF3 gene annotation file was not available for that assembly at the time of analysis. For reference-based annotation context, four additional fungal genomes were included together with the CM and CZ reference genomes: *Cercospora beticola* strain Cb09-40 (GCF_033473495.1) [43], *Cercospora kikuchii* strain MAFF 305040 (GCF_019650295.1) [44], *Pseudocercospora fijiensis* strain CIRAD86 (GCF_000340215.1) [45], and the outgroup *Zymoseptoria tritici* strain IPO323 (GCF_000219625.1) [46].

Orthologous relationships among the proteomes of these six species were inferred using OrthoFinder v3.0.1b1 [47]. Pairwise sequence similarity searches were performed with DIAMOND v2.1.10 in BLASTP mode using the “more sensitive” setting and default parameters otherwise [48]. Orthogroup clustering was then conducted with the MCL algorithm [49]. The resulting orthogroups provided reference-based functional context for grouping expressed transcripts and identifying putative orthologous relationships among reference-annotated loci. In this study, “CM-reference-specific” and “CZ-reference-specific” denote genes or orthogroups detected only in the SCOH1-5 or CMW25467 v3 reference annotation, respectively; these categories describe the two public reference genomes and are not species-wide classifications of the Jilin isolates. Reference-based orthogroup classifications, member proteins, and copy-number summaries used in the downstream analyses are provided in Table S2.

A reference-species phylogeny was constructed from 4929 single-copy orthogroups identified by OrthoFinder across the six public fungal reference genomes, with each orthogroup containing one ortholog from each genome. The amino acid sequences within each single-copy orthogroup were aligned separately using MAFFT v7 [32], and the resulting alignments were concatenated into a supermatrix using AMAS [34]. A maximum-likelihood phylogeny was then inferred using IQ-TREE v3.0.1 [35]. The resulting tree established the phylogenetic context of the six public reference genomes used for reference-based annotation and was not used to infer gene-content evolution or selection in the Jilin isolates.

### 2.6. Functional Annotation

Comprehensive protein domain and family prediction was performed using InterProScan v5.73-104.0 with all bundled databases (e.g., Pfam, SMART, PANTHER), retaining associated Gene Ontology (GO) terms and pathway mappings [50]. KEGG Orthology assignments were generated with KofamKOALA using the KOfam HMM library (v2024.03) and its built-in adaptive score thresholds [51]. Broad-scale functional annotation was conducted with eggNOG-mapper v2.1.12 (DIAMOND --sensitive; taxonomic scope = 4751, Fungi) [52]. The results were post-filtered using the following thresholds: E-value ≤ 1 × 10^−5^, bit score ≥ 60, percent identity ≥ 50%, and both query and subject coverage ≥ 60%. Pathogenicity-related genes were annotated via DIAMOND BLASTP searches against PHI-base v5.9 and the NCBI RefSeq fungi database (E-value ≤ 1 × 10^−5^), retaining only the best hit per query [53,54]. Proteases were identified by DIAMOND BLASTP search against the MEROPS database (E-value ≤ 1 × 10^−5^; percent identity ≥ 30%).

Carbohydrate-active enzymes (CAZymes) were annotated using the dbCAN2 pipeline: first, HMMER v3.0 hmmscan was used to search the dbCAN-HMM database (v13), filtering for domain E-value ≤ 1 × 10^−5^ and HMM score ≥ 30 [55]; DIAMOND BLASTP was then used to search against the CAZy database (v2024.07) with an E-value ≤ 1 × 10^−5^ and percent identity ≥ 30% [56]. The intersection of hits from both methods was considered as high-confidence cell wall–degrading enzymes (CWDEs).

Genes associated with cercosporin biosynthesis were identified using the CTB1–CTB12 protein sequences from *C. beticola* as queries in BLAST+ v2.14.0 using blastp to search against the proteomes of *C. zeae-maydis* and *C. zeina* [57]. Putative orthologs were assigned based on reciprocal best hits meeting the thresholds of E-value ≤ 1 × 10^−5^, sequence coverage ≥ 70%, and percent identity ≥ 70%.

### 2.7. Prediction of Candidate Effector Proteins

To identify candidate secreted effectors, a bioinformatic pipeline was applied to the predicted proteomes of *C. zeae-maydis* (CM) and *C. zeina* (CZ). First, all protein sequences were extracted from the genome annotations using gffread v0.12.7. An initial secretome was defined by predicting N-terminal signal peptides with SignalP 6.0 [58]. To remove potentially membrane-anchored proteins, the resulting set was further filtered with TMHMM v2.0 [59]. Any sequence containing one or more predicted transmembrane helices was excluded, yielding a final set of candidate secreted proteins. Putative effector proteins were then predicted from this secretome using EffectorP 3.0, which classifies candidates as cytoplasmic, apoplastic, or dual-localization effectors [60]. To support reference-guided annotation of putative orthologous candidate effectors between the two reference annotations, the directly predicted effector set was further expanded using orthology information. Specifically, when a protein in either CM or CZ was predicted to contain a signal peptide and was classified as an effector by EffectorP 3.0, its corresponding orthologous protein in the other species was also retained as an orthology-supported candidate effector, if present. Candidate effector proteins whose corresponding genes were not expressed in any sampled condition were removed from downstream expression analyses. The subcellular localization of these predicted effectors was inferred using DeepLoc2 v1.0.0 [61].

### 2.8. Statistical Analysis

All statistical analyses were performed in R as exploratory analyses based on the independently processed pooled samples collected during the single controlled inoculation time course. Differential expression analysis was conducted using DESeq2 to identify transcripts showing count-supported post-inoculation expression changes within each isolate dataset. For each isolate, post-inoculation time points were compared with the corresponding 0 dpi baseline using raw read counts after low-expression filtering. Transcripts with FDR < 0.05 and |log_2_ FoldChange| > 1 were retained as exploratory differentially expressed transcript candidates.

Differences in pathogen-derived read mapping rates between CM and CZ at each post-inoculation time point were assessed using Welch’s *t*-test on log_10_-transformed mapping percentages, calculated as log_10_ (Pathogen mapping rate + 0.01), to reduce the influence of heteroscedasticity in low-percentage data. *p*-values from time-point-wise comparisons were adjusted using the Benjamini–Hochberg method. These adjusted comparisons summarize variation among the independently processed pooled samples collected for each isolate and time point.

For transcriptome-level quality assessment, principal component analysis (PCA) was performed separately for CM and CZ using normalized pathogen gene expression profiles. Raw count matrices were normalized using the variance-stabilizing transformation implemented in DESeq2, and PCA was performed in R based on normalized expression values. Complete DESeq2 results and the associated GO-enrichment and functional-annotation datasets are provided in Tables S3–S9.

### 2.9. RT-qPCR Validation of Selected Candidate Genes

Quantitative reverse-transcription PCR (RT-qPCR) was performed to validate selected colonization-associated expression patterns identified by RNA-seq. Representative genes associated with cell wall-degrading enzymes, candidate effectors, protease-related functions, and lipid metabolism were selected, including GH11, GH5, Ecp6-like, Nis1-like, NPP1-like, A1 aspartic protease-related, and fatty acid β-oxidation-associated candidates. Host-associated leaf samples collected at 0, 1, 3, and 6 days post-inoculation (dpi) were analyzed, with three independently processed pooled samples for each isolate and time point.

Total RNA was extracted using the Ultrapure RNA Kit with DNase I (CW0597S, CWBIO, Taizhou, China) according to the manufacturer’s instructions. For each reverse-transcription reaction, 2 μg of total RNA was used. First-strand cDNA was synthesized using Oligo(dT)18 Primer (3806, Takara, Shiga, Japan), Reverse Transcriptase M-MLV (RNase H−; 2641A, Takara, Shiga, Japan), Cloned Ribonuclease Inhibitor (2313A, Takara, Japan), dNTP Mixture (4019, Takara, Shiga, Japan), and the supplied reaction buffer in a final volume of 20 μL. Reverse transcription was performed at 42 °C for 60 min. The resulting cDNA preparations were subsequently diluted five-fold with nuclease-free water before qPCR analysis.

qPCR was performed using SuperStar Universal SYBR Master Mix (CW3360M, CWBIO, Taizhou, China) on a Bio-Rad CFX Duet Real-Time PCR System (Bio-Rad, Hercules, CA, USA). Each 20 μL reaction contained 10 μL of 2× SuperStar Universal SYBR Master Mix, 0.4 μL of each primer (10 μM), 2 μL of diluted cDNA, and 7.2 μL of nuclease-free water. The cycling program consisted of 95 °C for 30 s, followed by 40 cycles of 95 °C for 5 s and 60 °C for 30 s. Melting-curve analysis was performed from 65 °C to 95 °C to assess amplification specificity. Gene-specific primers are listed in Table S10.

The same pooled-sample design described for RNA-seq sampling was used for RT-qPCR, with three independently processed pooled samples for each isolate and time point. β-Tubulin was used as the internal reference gene. For statistical comparison of RT-qPCR data between CM and CZ, the three pooled-sample ΔCt values for each isolate and time point were used as the statistical observations, whereas relative expression values were used for plotting. For each gene, a linear model including Isolate, Time, and their interaction was fitted using ΔCt as the response variable. Pairwise comparisons between CM and CZ were performed within each time point using estimated marginal means, with *p*-values adjusted within each gene using the Sidak method. The resulting adjusted *p*-values were used to evaluate isolate-associated CM–CZ differences at the same time point.

To compare the temporal expression trends obtained by RNA-seq and RT-qPCR, RNA-seq expression was summarized as the mean FPKM and standard deviation of the three independently processed pooled RNA-seq samples for each selected candidate, isolate, and time point. For each pooled sample, relative expression was calculated using the 2^−ΔΔCt^ method. RT-qPCR expression was then summarized as the mean across the three independently processed pooled samples for each selected candidate, isolate, and time point. For paired time-course visualization, RNA-seq mean FPKM values were displayed as bars with standard-deviation error bars, whereas mean RT-qPCR relative expression values were displayed as lines without error bars. Because FPKM and RT-qPCR relative expression have different measurement scales, separate y-axes retaining their original units were used to facilitate visual comparison of temporal patterns rather than absolute expression magnitudes. For each selected candidate within each isolate, a descriptive Pearson correlation coefficient (*r*) was calculated from the paired mean RNA-seq FPKM and mean RT-qPCR relative expression values at 1, 3, and 6 dpi. The 0 dpi values were displayed as the experimental baseline but were excluded from the correlation calculation.

## 3. Results

### 3.1. Identification, Symptom Development, and Pathogen-Derived Transcript Representation of C. zeae-maydis and C. zeina Isolates During Maize Leaf Colonization

A multilocus phylogenetic analysis based on the concatenated ITS, TEF1, ACT, CAL, and HIS3 dataset was first performed to confirm the taxonomic identity of the two Jilin isolates used in this study. The maximum-likelihood phylogeny, rooted with *Pseudocercospora griseola*, placed the CM isolate within the *C. zeae-maydis* reference group, clustering closely with *C. zeae-maydis* strain SCOH1-5 and other *C. zeae-maydis* reference sequences. In contrast, the CZ isolate clustered with *C. zeina* strain CMW25467 and other *C. zeina* reference sequences (Figure S2A). Branch lengths are shown in substitutions per site.

Pairwise p-distance analysis based on the same concatenated alignment further supported the phylogenetic placement. The key pairwise comparisons are summarized in Figure S2B, including the number of nucleotide differences, the number of compared sites, p-distance values, and percentage divergence. The CM isolate was much closer to *C. zeae-maydis* SCOH1-5 than to *C. zeina* CMW25467, with p-distances of 0.0068 and 0.0581, respectively. Conversely, the CZ isolate was closer to *C. zeina* CMW25467 than to *C. zeae-maydis* SCOH1-5, with p-distances of 0.0097 and 0.0555, respectively. The distance between CM and CZ was 0.0600, comparable to the divergence between the two reference species sequence sets, which was 0.0649. These results support the assignment of the CM and CZ Jilin isolates to *C. zeae-maydis* and *C. zeina*, respectively.

**Figure 1:**
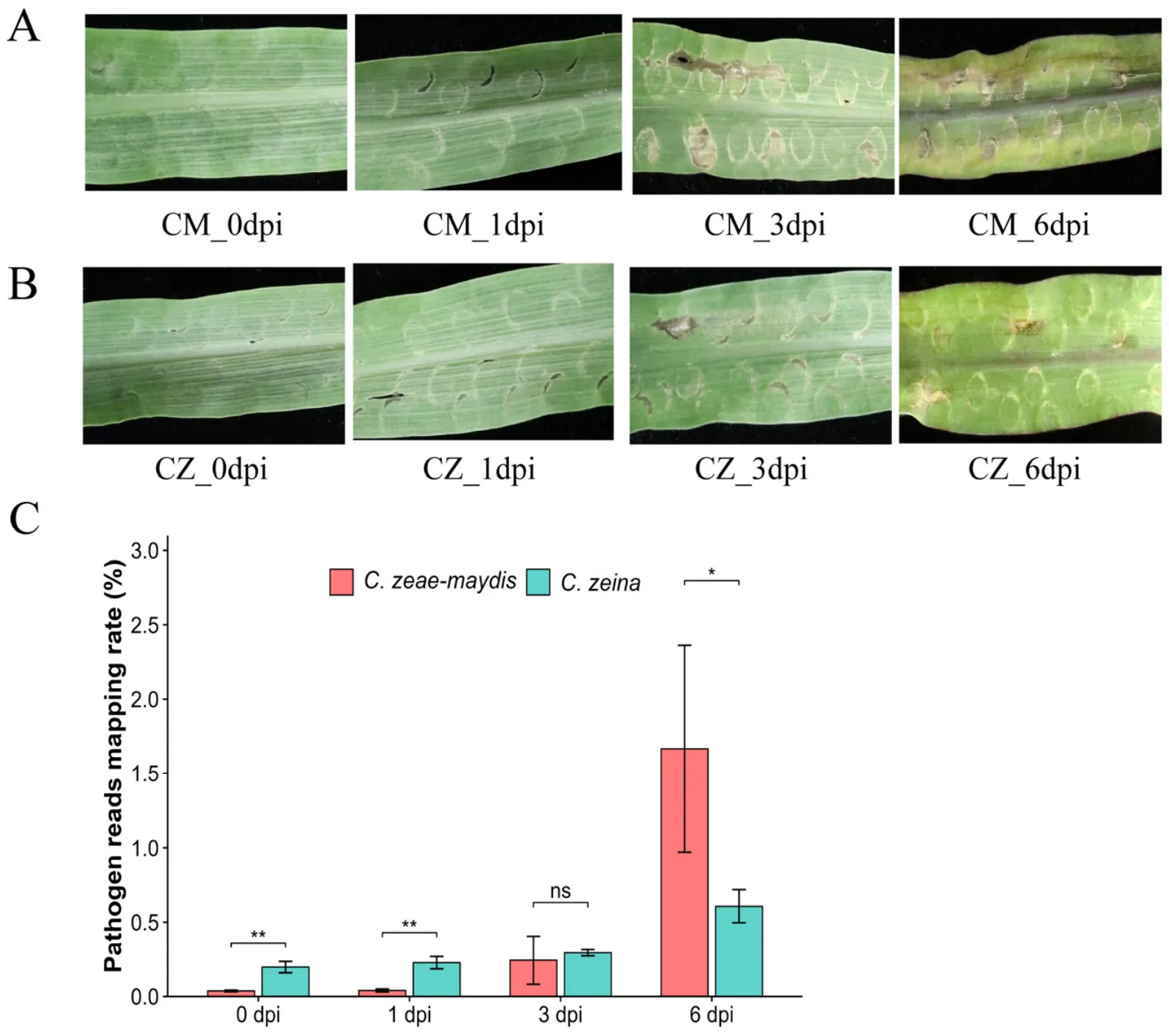
Disease progression and pathogen read-mapping rates during maize infection by *C. zeae-maydis* and *C. zeina*. Representative disease symptoms on maize B73 seedlings inoculated with *C. zeae-maydis* (**A**) or *C. zeina* (**B**) were recorded at the indicated time points post-inoculation to document lesion development during infection. (**C**) Pathogen-derived read mapping rates in mixed host–pathogen RNA-seq libraries at 0, 1, 3, and 6 dpi. Bars represent mean ± SD for the three independently processed pooled samples from the single controlled inoculation experiment. Raw pathogen mapping percentages are shown for visualization. Statistical comparisons between CM and CZ at each time point were performed using Welch’s *t*-test on log_10_-transformed mapping percentages, followed by Benjamini–Hochberg correction. Asterisks indicate differences supported by exploratory comparisons between CM and CZ at the same time point (* adjusted *p* < 0.05, ** adjusted *p* < 0.01; ns, no significance). The proportion of pathogen-mapped reads was used to summarize pathogen-derived transcript representation in the mixed host–pathogen RNA-seq libraries. This metric should be interpreted as an indicator of pathogen-derived transcript representation rather than a direct measurement of pathogen abundance or population size.

Maize B73 seedlings were then inoculated with spore suspensions of the representative *C. zeae-maydis* Jilin isolate or *C. zeina* Jilin isolate using a needleless syringe-assisted inoculation method. Leaf tissues were collected at 0, 1, 3, and 6 dpi for pathogen-focused RNA-seq analysis. During the RNA-seq sampling window, no visible symptoms were observed at 0 or 1 dpi for either isolate. By 3 dpi, initial symptoms appeared in leaves inoculated with both CM and CZ. At 6 dpi, CM-inoculated leaves showed extensive necrosis and a larger diseased area, whereas CZ-inoculated leaves showed visible but more limited necrotic symptoms (Figure 1A,B). These observations indicate that, under this controlled inoculation system, the CM isolate showed faster visible symptom development than the CZ isolate.

To confirm that the controlled inoculation system could reproduce typical gray leaf spot symptoms, inoculated leaves were also monitored beyond the RNA-seq sampling window. At later stages, CM-inoculated leaves at 15 dpi and CZ-inoculated leaves at 20 dpi developed rectangular gray leaf spot lesions delimited by leaf veins (Figure S1E,F). These observations support that both isolates were able to colonize maize leaves and reproduce characteristic GLS lesions under the assay conditions.

The two isolates also showed distinct in vitro culture phenotypes. On PDA medium after 8 days of growth, CM formed a larger white to pale mycelial colony, whereas CZ formed a smaller and more compact colony. On maize leaf powder sporulation medium, CM produced abundant pinkish to pale sporulating colonies after fungal biomass was spread onto the medium surface, whereas CZ produced smaller and more discrete pale colonies under the same conditions (Figure S1A–D). These observations provide additional phenotypic documentation of the representative isolates used in this study.

Pathogen-focused RNA-seq analysis was then performed using inoculated maize leaf samples. The proportion of pathogen-derived reads, calculated as pathogen-mapped reads relative to total reads, increased over time for both isolates (Figure 1C; Table 1). At 0 and 1 dpi, exploratory comparisons based on log_10_-transformed values with Benjamini–Hochberg correction indicated higher pathogen-derived read mapping rates in CZ than in CM. This early difference was consistent with the higher inoculum concentration used for CZ and supports the interpretation that early pathogen-derived read representation was influenced by inoculum input. By 3 dpi, pathogen-derived read proportions were comparable between the two isolates, and no difference was supported by the adjusted comparison. By 6 dpi, the proportion of CM-derived reads reached 1.67%, approximately 2.7-fold higher than the 0.61% observed for CZ, and this difference was supported by the adjusted comparison of log_10_-transformed values. This later increase in CM-derived transcript representation was consistent with the stronger symptom development observed for CM at the later sampled time points.

Because pathogen-mapped read proportions can be influenced by sampling, sequencing depth, alignment efficiency, host tissue composition, and reference genome divergence, this metric was interpreted only as an indicator of pathogen-derived transcript representation in mixed host–pathogen RNA-seq libraries, rather than as a direct measurement of pathogen abundance or population size. In Figure 1C, raw mapping percentages are shown for visualization, whereas statistical comparisons were performed using log_10_-transformed values.

To evaluate the consistency of the pathogen-focused RNA-seq datasets, PCA was performed separately for CM and CZ using normalized pathogen gene expression profiles. For both isolates, independently processed pooled samples from the same condition generally clustered closely, indicating consistency among independently processed pooled samples within the single controlled inoculation experiment (Figure S3). In vitro samples were clearly separated from host-associated samples along PC1, indicating a major transcriptional shift from culture growth to host-associated growth. Post-inoculation samples also showed time-dependent separation, particularly between early and later sampling points. These results support the suitability of the datasets for downstream differential expression and temporal expression analyses. For reference-based annotation context, the phylogenetic relationships among the six public fungal reference genomes were reconstructed from 4929 single-copy orthogroups (Figure S4). This reference-species tree provides the phylogenetic framework for the orthogroup-based organization of mapped transcripts used in the subsequent analyses.

### 3.2. Shared and Isolate-Associated Transcriptional Programs During Maize Infection by Two Isolates of Different Species

Based on pathogen-derived reads from the inoculated leaf RNA-seq datasets, differential expression analysis was performed separately for each Jilin isolate by comparing each post-inoculation time point (1, 3, and 6 dpi) with the corresponding 0 dpi baseline using raw read counts in DESeq2 after low-expression filtering. Genes showing low expression at 0 dpi but elevated expression at later time points and meeting the exploratory DESeq2 criteria were classified as candidate post-inoculation-induced transcripts. Under these criteria, 801 upregulated reference-annotated transcripts were identified in the CM dataset and 1691 in the CZ dataset, with full DESeq2 statistics and functional annotations provided in Table S3.

GO enrichment analysis showed that both isolates activated functions related to translation, ribosome-related processes, carbohydrate catabolism, and redox processes under host-associated conditions, indicating that enhanced protein synthesis and metabolic activity were common features of maize leaf colonization (Figure S5; Table S4). Despite these shared responses, the two isolates also showed differences in the functional composition of their upregulated transcript sets. Upregulated transcripts in the CM dataset were more strongly enriched in peptide and amide metabolism, ATP metabolism, and carbohydrate transport and degradation, whereas those in the CZ dataset were more prominently enriched in carbon metabolism, energy production, and oxidative stress-related functions. Gene identities in the following analyses correspond to reference-guided annotations of transcripts detected in the two Jilin isolates.

### 3.3. Host-Associated Expression of Reference-Annotated Cell Wall-Degrading Enzyme Candidates

Using dbCAN2 annotation of the two public reference proteomes, we defined a reference-annotated CWDE-associated CAZyme candidate set to support interpretation of mapped Jilin isolate transcripts (Table S5). Among these reference candidates, transcripts corresponding to 1238 CM-reference loci and 1155 CZ-reference loci were detected at one or more post-inoculation time points, indicating that the CM transcript dataset represented a slightly larger set of reference-annotated CWDE-associated loci under the sampled conditions.

**Figure 2:**
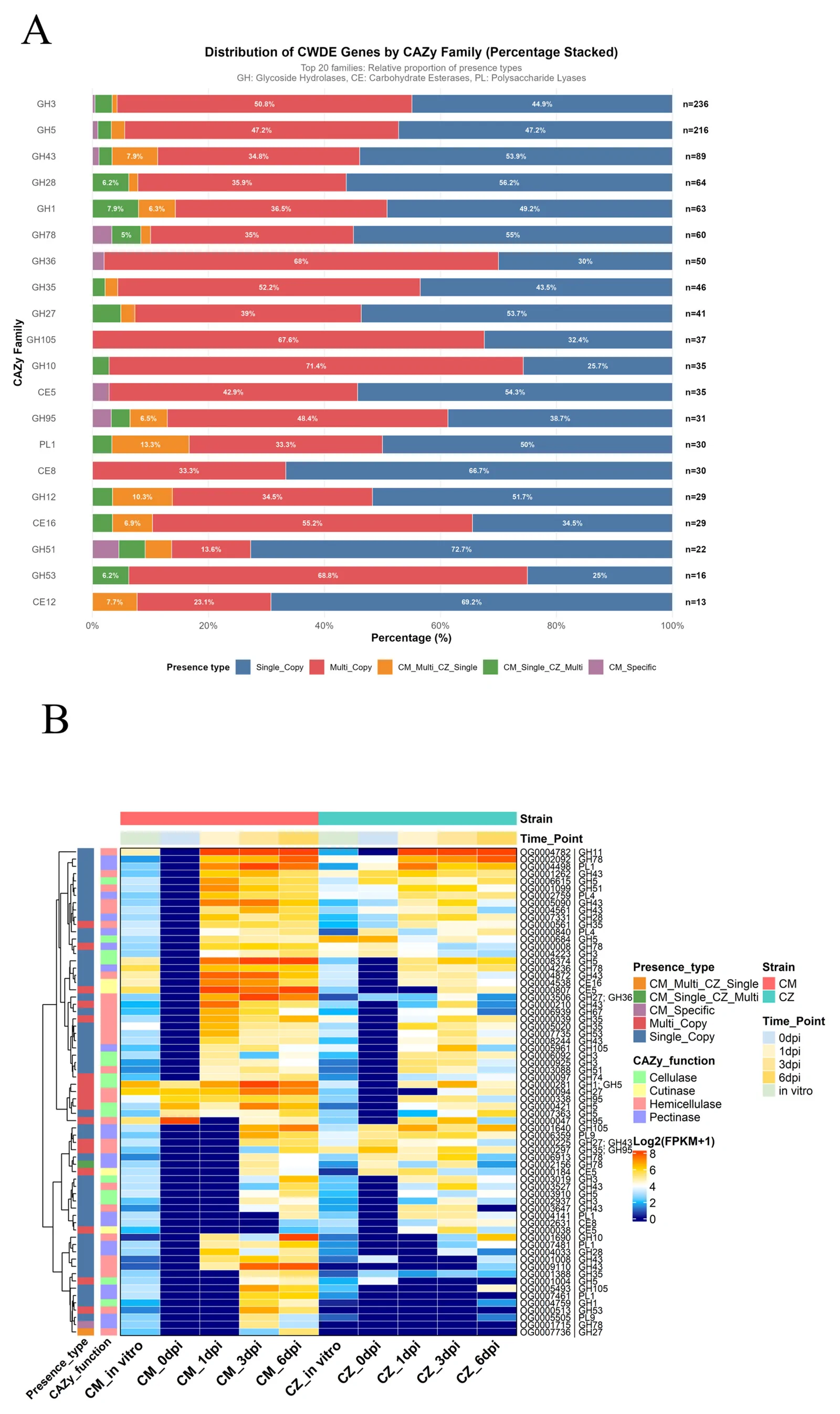
Reference-guided annotation and expression patterns of CWDE-associated candidate transcripts in the two Jilin isolates. (**A**) Distribution of expressed reference-annotated CWDE-associated candidates across selected CAZy families and reference-based orthogroup categories. These annotations were assigned by mapping Jilin isolate-derived reads to the *C. zeae-maydis* SCOH1-5 and *C. zeina* CMW25467 v3 reference gene models. Orthogroup categories in this panel provide reference-based annotation context and do not establish gene presence/absence or copy-number differences in the Jilin isolate genomes. CM_Specific denotes reference orthogroups represented only in the SCOH1-5 annotation. (**B**) Heatmap showing expression patterns of selected post-inoculation-induced CWDE-associated candidate transcripts in CM and CZ. Genes showing low expression in in vitro samples and at 0 dpi, but elevated expression at 1, 3, or 6 dpi were selected. Expression levels are shown as log_2_ (FPKM + 1) to visualize temporal trends; differential-expression statistics based on DESeq2 analysis of raw counts are provided in Table S3.

The reference-annotated CWDE candidates were classified mainly as cellulases, hemicellulases, pectinases, and cutinases, with cellulase-related candidates representing the largest functional category in both reference annotations (Figure S6). At the CAZy family level, expressed CWDE-associated transcripts mapped to GH, CE, and PL classes, including families involved in degradation or modification of cellulose, hemicellulose, pectin, and cutin, such as GH5, GH3, GH43, GH28, GH35, GH53, PL1, and CE8 (Figure 2A). Orthogroup categories shown in Figure 2A were used to describe the reference-based annotation context for expressed CWDE-associated transcripts. Although the two transcript datasets shared the major CWDE-associated CAZy classes, the relative distributions of reference orthogroup categories varied among individual CAZy families (Figure 2A).

### 3.4. Reference-Guided Candidate Effector Annotation and Host-Associated Expression

**Figure 3:**
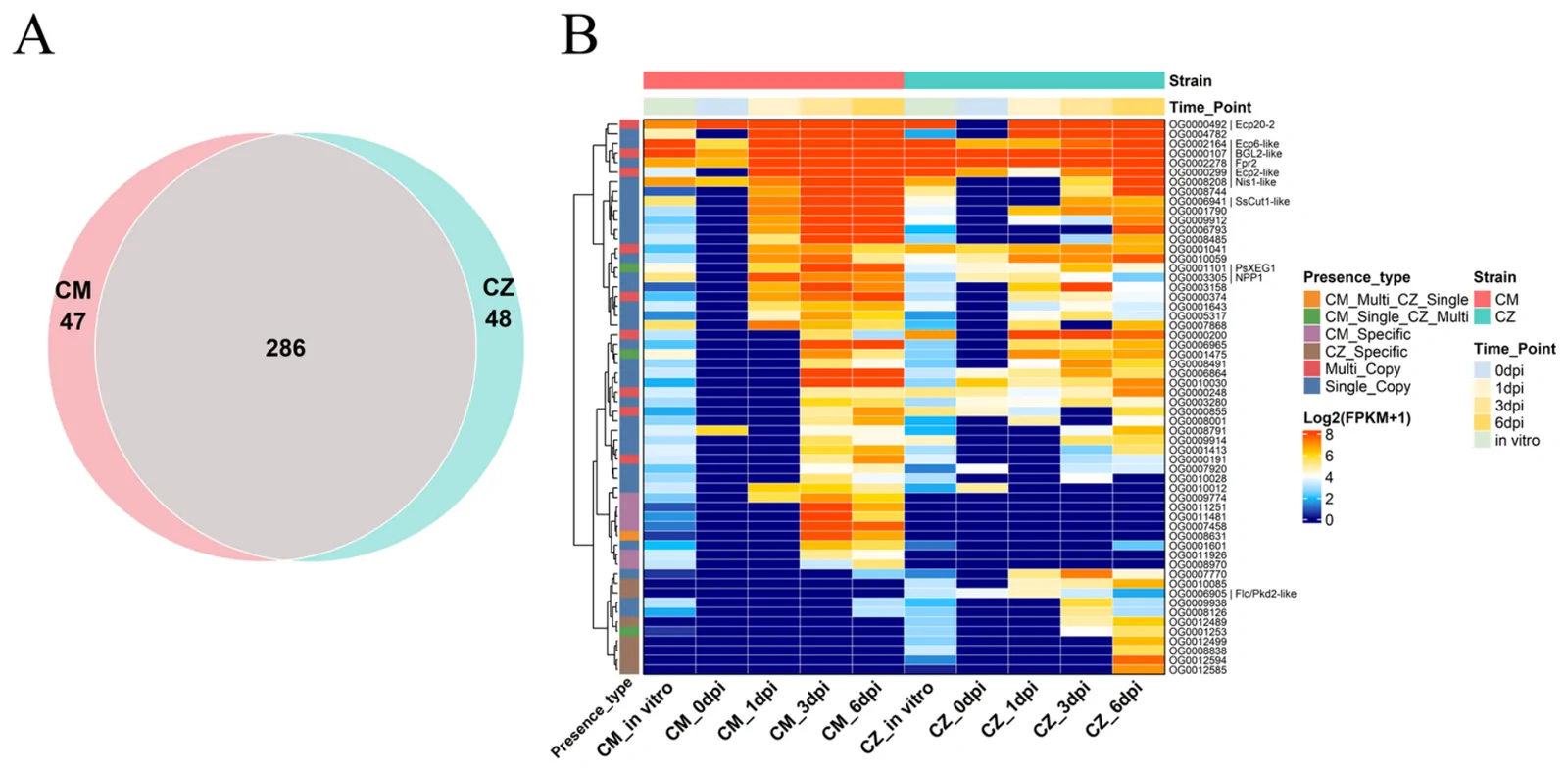
Reference-guided candidate effector annotation and expression patterns during maize leaf colonization by the two Jilin isolates. (**A**) Summary of reference-annotated candidate effector proteins used to support putative functional annotation of mapped transcripts. These categories are based on the *C. zeae-maydis* SCOH1-5 and *C. zeina* CMW25467 v3 reference annotations and do not demonstrate effector gene presence or absence in the Jilin isolate genomes. (**B**) Expression profiles of representative effector-associated candidate transcripts at different post-inoculation time points during maize leaf colonization. Expression levels are shown as log_2_ (FPKM + 1) for visualization of temporal trends only; statistical inference of differential expression was based on DESeq2 analysis of raw counts. Expression differences are interpreted as candidate time-point-associated patterns under the controlled inoculation system and not as direct functional evidence of effector activity. CM_Specific and CZ_Specific denote reference orthogroups represented only in the SCOH1-5 and CMW25467 v3 annotations, respectively.

Reference-annotated candidate secreted effector-associated loci were identified from the *C. zeae-maydis* SCOH1-5 and *C. zeina* CMW25467 v3 annotations to support putative functional annotation of mapped Jilin isolate transcripts (Figure 3A; Table S6). After filtering for candidates with expression support in at least one sampled condition, 333 CM-reference and 334 CZ-reference candidate effector-associated loci were retained for transcript-level analysis. Of these, 286 candidates had orthogroup counterparts in both reference annotations, whereas 47 and 48 candidates were labeled in Figure 3A as CM-specific and CZ-specific, respectively. The abbreviated labels denote CM-reference-specific and CZ-reference-specific candidates within the paired public reference annotations.

Integration of PHI-base and NCBI nr homology searches with InterPro/Pfam annotation and DeepLoc2 subcellular localization prediction identified multiple candidate effectors and pathogenicity-related proteins homologous to previously characterized virulence-associated factors. Representative candidates identified by PHI-base-guided annotation and homology searches are summarized in Table 2. Among these, several homologs of canonical fungal effector families were represented, including Ecp6-like, Ecp2-like, Ecp20-2, Nis1-like, and NPP1-like proteins, as well as cell wall degradation-associated effectors such as PsXEG1-like, SsCut1-like, and BGL2-like proteins (Figure S7). Expression profiling further showed that a subset of genes encoding candidate effectors was weakly expressed in in vitro cultures and at 0 dpi, but strongly induced at 1, 3 or 6 dpi, indicating host-associated induction under the controlled inoculation system (Figure 3B). At the sampled chronological post-inoculation time points, the CM isolate exhibited earlier and stronger expression of several candidate virulence-associated effector homologs, including genes encoding Ecp6-like, Ecp2-like, Ecp20-2, Nis1-like, PsXEG1-like, and SsCut1-like proteins, whereas the corresponding genes in CZ were generally induced later or at lower levels (Figure 3B). These early and highly expressed effector homologs may be associated with the faster symptom development and higher pathogen-derived transcript proportion observed for the CM isolate under the controlled inoculation system. Because CM and CZ may not have reached equivalent physiological infection stages at the same dpi, these differences should be interpreted as chronological time-point-associated expression patterns rather than strictly stage-matched comparisons. In addition, the gene encoding the BGL2-like protein was actively expressed throughout infection in both pathogens.

In addition to canonical effector homologs, several secreted proteins with enzymatic or accessory virulence-related annotations showed reference-specific or isolate-associated expression patterns during maize leaf colonization (Table S6). Representative shared but CM-biased examples included a GH28 polygalacturonase encoded by OG0001980 (CM: KAF2216020; CZ: KAM3425002.1) and a secreted GH11 xylanase encoded by OG0004782 (CM: KAF2208979; CZ: KAM3424045.1). Both genes were strongly induced in CM during post-inoculation stages, whereas their CZ orthologs were expressed at lower levels (Table S6). One candidate only present in the *C. zeina* CMW25467 v3 annotation, OG0006905 (KAM3421994.1), was annotated as an Flc/Pkd2-like protein carrying an ML-like domain and showed sequence similarity to the fungal ion channel FlcA from Aspergillus fumigatus, a PHI-base entry associated with loss of pathogenicity. The corresponding transcript was upregulated during maize leaf colonization in the CZ dataset (Figure S7; Table S6). Because this candidate was defined from the reference annotation, its presence and structure in the Jilin CZ genome remain to be confirmed.

### 3.5. Expression Patterns of Genes Associated with Cercosporin Biosynthesis During Maize Leaf Colonization

**Figure 4:**
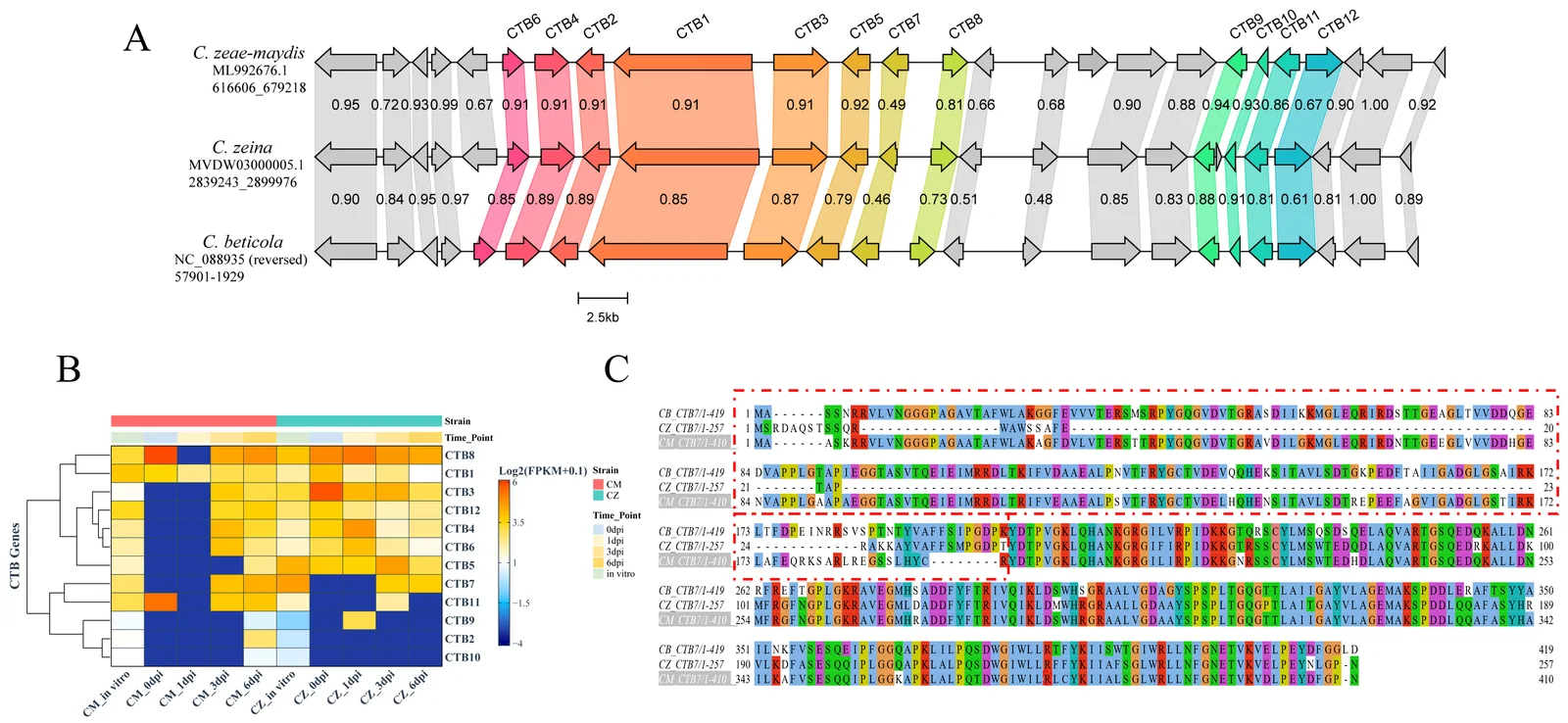
Reference-based organization and expression profiles of genes associated with cercosporin biosynthesis during maize leaf colonization. (**A**) Reference-genome organization of genes associated with cercosporin biosynthesis in *C. zeae-maydis* SCOH1-5, *C. zeina* CMW25467 v3, and *C. beticola*. This panel defines the homologous gene set used for transcript-level analysis and provides genomic context for interpreting the expression of cercosporin-biosynthesis-associated genes. Colored arrows denote CTB homologs, matching colors indicate corresponding homologs across species, gray arrows denote neighboring genes, and arrow direction indicates gene orientation. The numerical values associated with the shaded connections indicate pairwise amino acid sequence identity between the linked homologous proteins. (**B**) Heatmap showing expression profiles of cercosporin-biosynthesis-associated transcripts in the representative *C. zeae-maydis* Jilin isolate (CM) and *C. zeina* Jilin isolate (CZ) based on reference-guided read mapping across in vitro and post-inoculation time points. Expression levels are shown as log_2_ (FPKM + 0.1) for visualization of temporal trends only; statistical inference of differential expression was based on DESeq2 analysis of raw counts. (**C**) Reference-based multiple sequence alignment of CTB7 homologs from *C. beticola*, *C. zeae-maydis* SCOH1-5, and *C. zeina* CMW25467 v3. The boxed region indicates an amino acid sequence segment present in the *C. beticola* and *C. zeae-maydis* CTB7 homologs but absent from the aligned *C. zeina* CTB7 homolog. The shorter CTB7 homolog in the *C. zeina* reference genome has been reported previously and is shown here as reference context for transcript annotation. Panels A and C are based on public reference sequences, whereas panel B presents transcript expression in the two Jilin isolates.

Homology searches using CTB1–CTB12 proteins from *Cercospora beticola* identified corresponding genes associated with cercosporin biosynthesis in the *C. zeae-maydis* SCOH1-5 and *C. zeina* CMW25467 v3 reference annotations (Figure 4A; Table S7). The reference-based CTB organization defined the homologous gene set used for transcript-level analysis and provided genomic context for interpreting cercosporin-biosynthesis-associated expression.

The primary analysis focused on the expression of genes associated with cercosporin biosynthesis during maize leaf colonization by the two Jilin isolates. CTB1 was expressed in both isolates across the sampled conditions, whereas several other genes associated with cercosporin biosynthesis showed time-dependent expression differences between CM and CZ. In particular, CTB3, CTB4, CTB5, CTB6, and CTB12 showed detectable expression across sampled conditions in the CZ dataset, while in the CM dataset these genes were lowly expressed or not induced at early sampled post-inoculation time points and became more clearly expressed at later time points (Figure 4B). CTB7 showed little or no expression at early post-inoculation stages and increased expression at later stages in both isolates. These expression profiles indicate that CTB-associated transcripts were detected during maize leaf colonization and that their temporal expression differed between the two Jilin isolates under the controlled inoculation system.

The CTB7 alignment provides reference-based context for the previously reported shorter *C. zeina* reference homolog (Figure 4C) [24]. The principal result of this analysis is the time-resolved expression of genes associated with cercosporin biosynthesis in the two Jilin isolates.

### 3.6. Host-Associated Expression of Reference-Annotated Lipid Metabolism-Associated Transcripts in the Two Jilin Isolate Datasets

**Figure 5:**
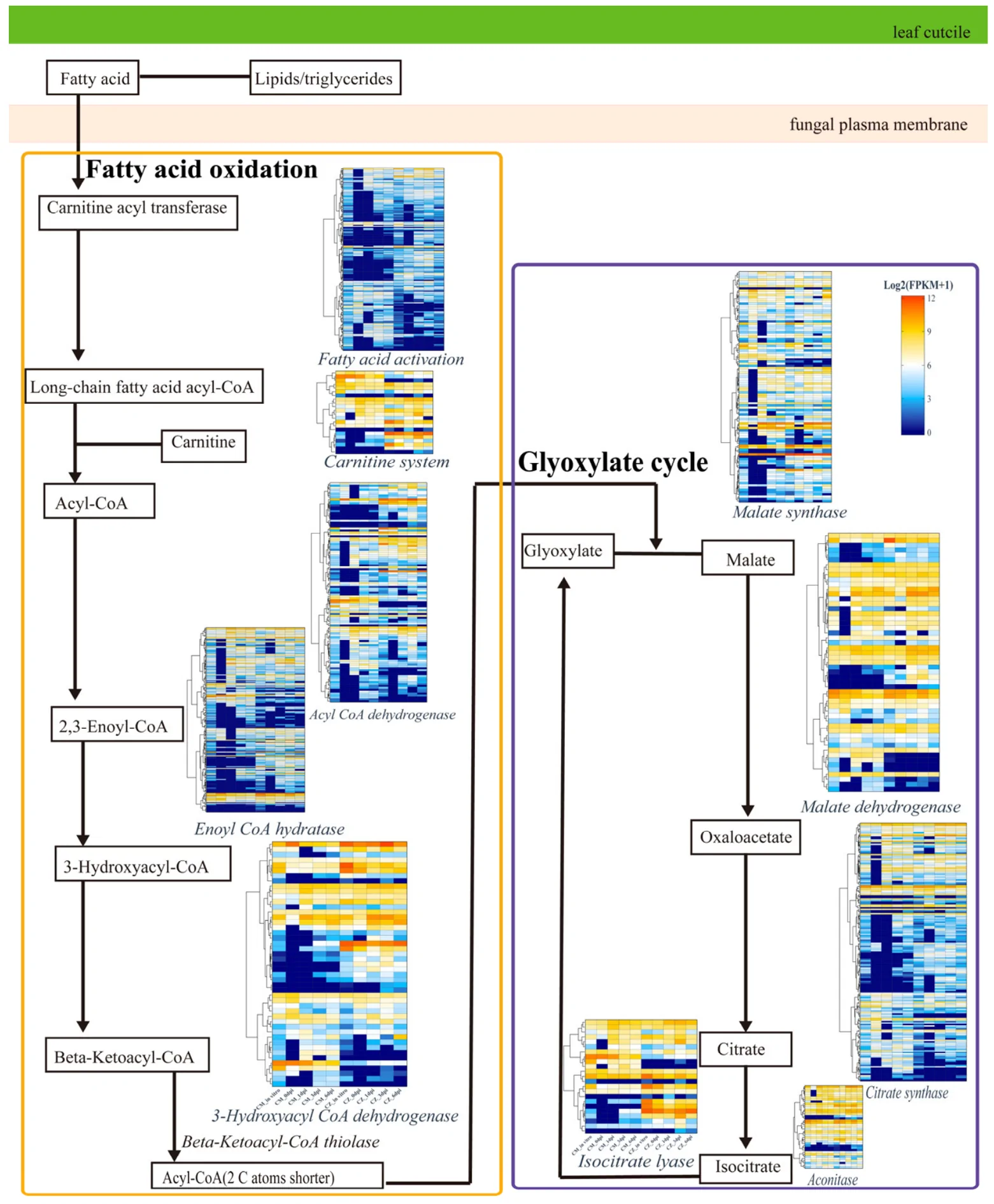
Expression profiles of candidate genes associated with fatty acid β-oxidation and the glyoxylate cycle in the two Jilin isolates. The heatmap shows reference-guided expression profiles of candidate lipid metabolism-associated transcripts during maize leaf colonization. Rows represent orthogroups and columns represent sampled conditions in CM and CZ. Expression levels are shown as log_2_ (FPKM + 1) for visualization of temporal trends only; statistical inference of differential expression was based on DESeq2 analysis of raw counts. Arrows indicate the direction of the depicted metabolic steps, whereas the orange and purple boxes delimit the fatty acid β-oxidation and glyoxylate-cycle modules, respectively.

Utilization of host-derived lipids can influence the colonization ability of phytopathogenic fungi. Based on reference-guided functional annotation using InterPro, Pfam, and KEGG, we identified candidate transcripts associated with fatty acid β-oxidation, the glyoxylate cycle, and related lipid metabolic processes in the two Jilin isolate RNA-seq datasets (Figure 5; Table S8). Several lipid metabolism-associated candidates were markedly induced under host-associated conditions. Transcripts corresponding to OG0000035, OG0008358, and OG0004122, annotated as fatty acid β-oxidation-related genes, were strongly induced after inoculation in both CM and CZ. Similarly, transcripts corresponding to OG0000374, OG0000678, and OG0001462, predicted to be involved in the glyoxylate cycle, showed host-associated upregulation in both isolates. Some candidates showed isolate-associated differences in temporal expression; for example, transcripts from OG0008389 showed preferential induction during maize leaf colonization in CM, whereas those from OG0001749 showed preferential induction in CZ. Together, these results indicate that lipid catabolic pathways were transcriptionally activated under host-associated conditions, with some candidates displaying isolate-associated expression patterns. Because these identities were assigned by mapping to reference gene models, they should be interpreted as putative functional annotations of expressed transcripts.

### 3.7. Host-Associated Expression of Reference-Annotated Protease-Related Transcripts

**Figure 6:**
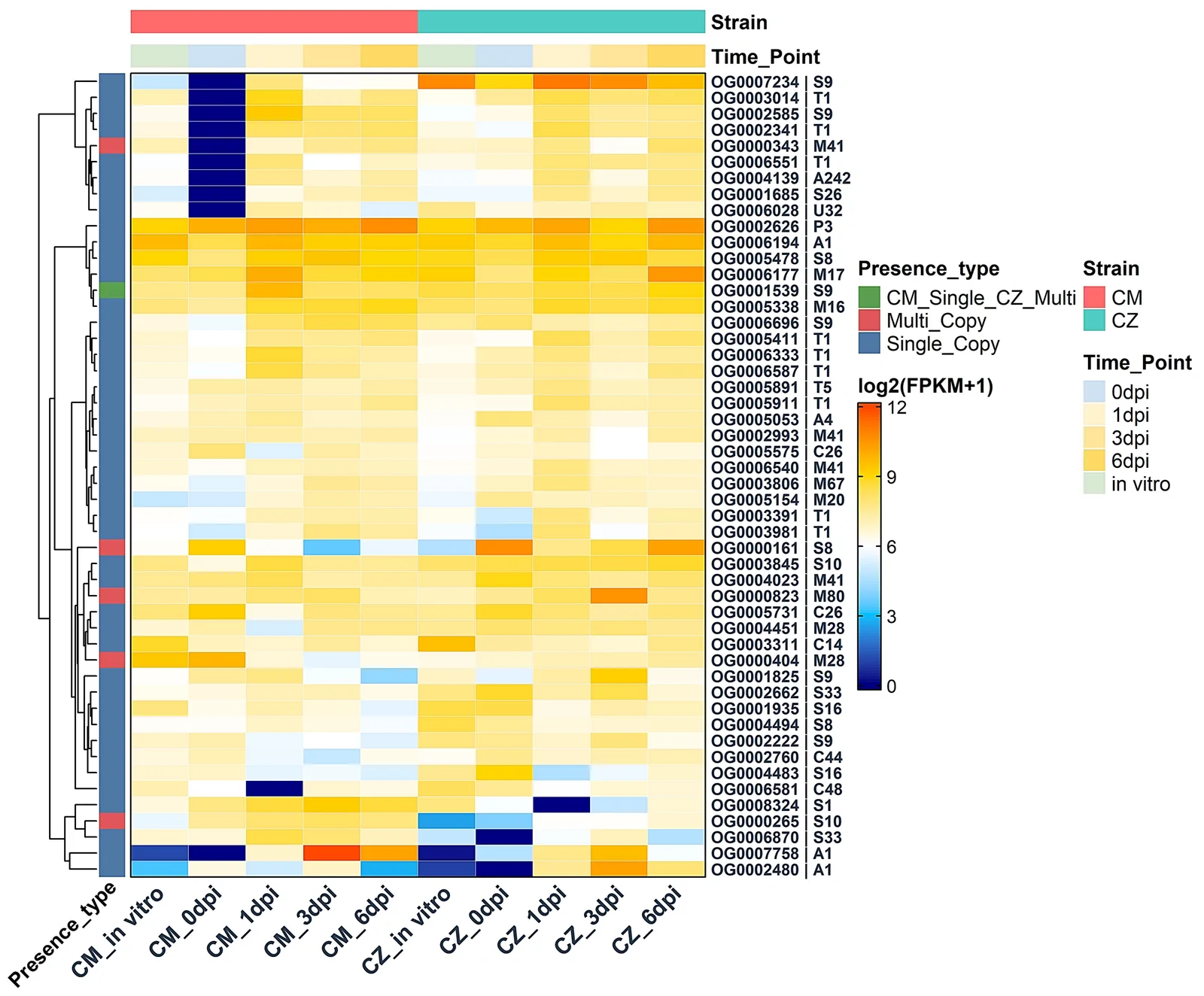
Expression profiles of selected highly expressed protease-related candidate transcripts in the two Jilin isolates. The heatmap shows FPKM-based expression profiles of the 50 most highly expressed protease-related genes across sampled conditions. Rows are labeled as Orthogroup | protease family, and color intensity reflects transcript abundance. The figure illustrates descriptive temporal expression patterns of selected protease-related candidate transcripts under the controlled inoculation system. Expression levels are shown as log_2_ (FPKM + 1) for visualization of temporal trends only; statistical inference of differential expression was based on DESeq2 analysis of raw counts.

Annotation against the MEROPS database identified reference-annotated protease and protease-related candidates in the *C. zeae-maydis* SCOH1-5 and *C. zeina* CMW25467 v3 reference annotations, which were used to interpret mapped transcripts from the two Jilin isolates (Figure 6; Table S9). These proteins spanned the major catalytic classes of fungal proteases, including serine, cysteine, aspartic, metalloproteases, threonine proteases, and glutamic proteases, as well as several protease-related homologs that retained protease-like domains but lacked conserved catalytic residues. Several representative orthogroups were classified into known protease families, including A1 aspartic proteases, M80 and M24A metalloproteases, and S8 subtilisin-like serine peptidases. For instance, A1 members included orthogroup OG0000147, which contains homologs with an Aspergillopepsin-like catalytic domain, and OG0007758, which contains homologs with a Pepsin-like domain. Metalloproteases included OG0005938 (M80) and OG0000995 (M24), the latter containing homologs with a creatinase/aminopeptidase-like domain and a MetAP2 active site. Multiple serine protease orthogroups were assigned to the S8 family, including OG0005478, OG0004884, and OG0004494. Interestingly, the genes corresponding to OG0007758 were expressed at low levels or not expressed in vitro or at 0 dpi in both Jilin isolate datasets, but were induced to high levels under host-associated conditions (Figure 6). These results indicate that mapped transcripts from both Jilin isolates included diverse protease-related candidates, with a subset of protease-associated transcripts, particularly aspartic protease-related candidates, showing host-associated induction during maize leaf colonization.

### 3.8. RT-qPCR Validation of Selected Colonization-Associated Candidate Genes

To provide independent transcript-level validation of selected RNA-seq-derived expression patterns, RT-qPCR was performed for representative genes associated with CWDEs, candidate effectors, lipid metabolism, and protease-related functions. The selected genes included GH5 (OG0008374) and GH11 (OG0004782) CWDE-associated candidates, Ecp6-like (OG0002164), Nis1-like (OG0008208), and NPP1-like (OG0003305) candidate effector homologs, a fatty acid β-oxidation-associated gene (OG0004476), and an A1 aspartic protease-related gene (OG0007758). Statistical comparisons were performed between CM and CZ within each time point using ΔCt values, whereas relative expression values were used for visualization.

**Figure 7:**
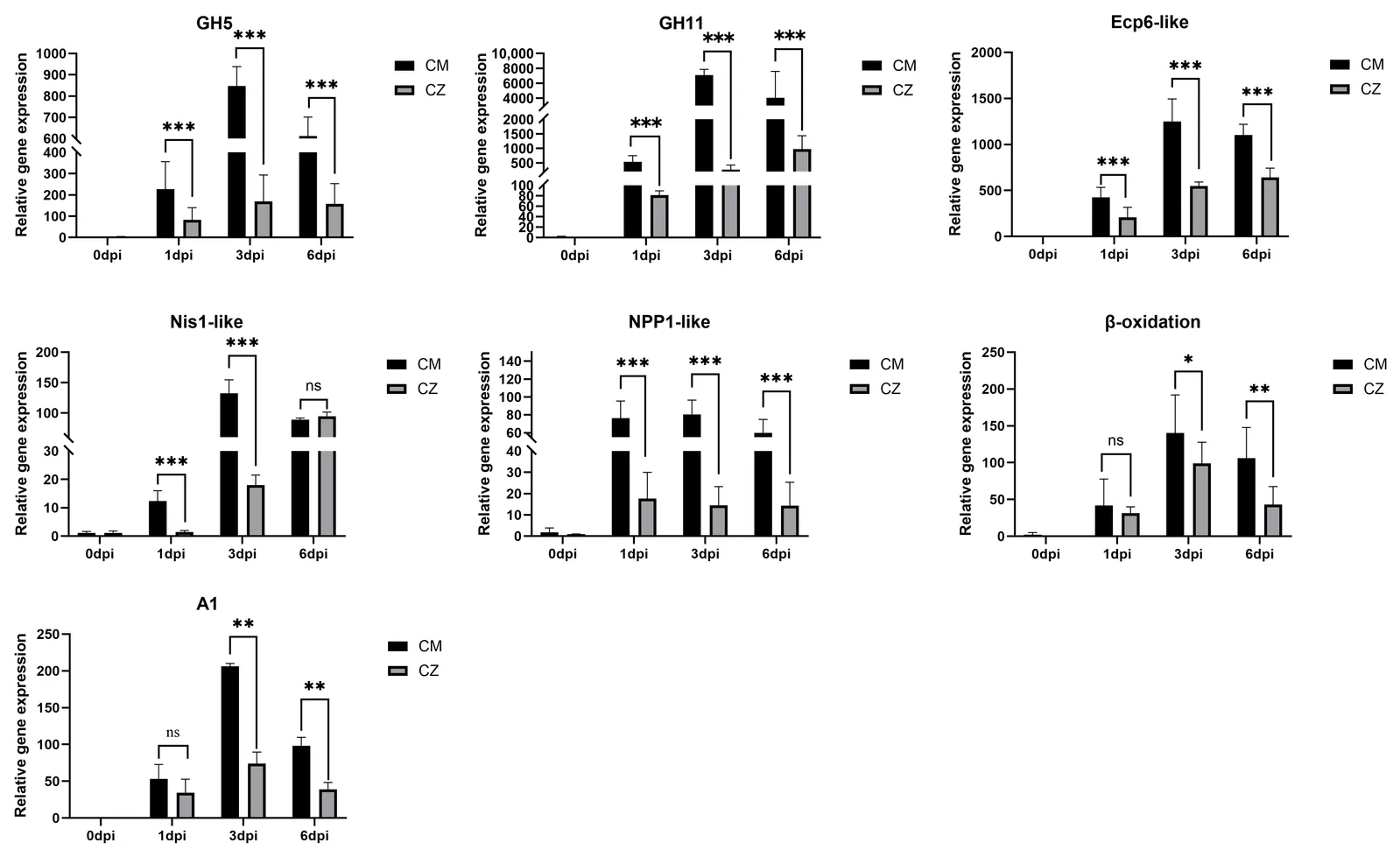
RT-qPCR validation of selected colonization-associated candidate genes in representative *C. zeae-maydis* and *C. zeina* isolates during maize leaf colonization. Relative expression patterns of selected genes associated with cell wall-degrading enzymes (GH5 and GH11), candidate effectors (Ecp6-like, Nis1-like, and NPP1-like), protease-related function (A1 aspartic protease-related gene), and lipid metabolism (fatty acid β-oxidation-associated gene) were examined at 0, 1, 3, and 6 dpi. Black bars indicate the representative *C. zeae-maydis* isolate (CM), and gray bars indicate the representative *C. zeina* isolate (CZ). Relative gene expression was calculated using the 2^−ΔΔCt^ method with β-Tubulin as the internal reference gene and the corresponding 0 dpi sample as the calibrator. Bars represent mean ± standard error across three independently processed pooled leaf samples. Each pooled sample was assayed in two technical qPCR reactions, which were averaged before sample-level statistical analysis. Exploratory RT-qPCR comparisons between CM and CZ were performed within each time point using ΔCt values as the statistical input, whereas relative expression values were used for plotting. Asterisks indicate CM–CZ differences supported by this analysis at the same time point after multiple-comparison adjustment within each gene (* *p* < 0.05, ** *p* < 0.01, *** *p* < 0.001; ns, no significance). These data provide transcript-level validation of selected RNA-seq-derived expression patterns for the selected candidates.

Overall, the RT-qPCR results supported post-inoculation expression changes for the selected candidates in CM and/or CZ and were broadly consistent with the RNA-seq-derived expression patterns within this experiment (Figure 7). For the CWDE-associated genes, GH5 and GH11 showed increased relative expression after inoculation and were expressed at higher levels in CM than in CZ at 1, 3, and 6 dpi in the RT-qPCR analysis. These results support stronger post-inoculation activation of selected CWDE-associated candidates in the CM isolate under the controlled inoculation conditions used here. Among candidate effector homologs, Ecp6-like and NPP1-like also showed higher expression in CM than in CZ at 1, 3, and 6 dpi in the RT-qPCR analysis. Nis1-like was strongly induced in CM at 1 and 3 dpi and showed higher expression in CM than in CZ at these two time points, whereas the CM–CZ difference was not supported at 6 dpi in this analysis. Thus, the RT-qPCR results support stronger CM-biased expression for selected effector-related candidates within this experiment, although this pattern was not observed for every gene at every time point.

For the representative fatty acid β-oxidation-associated gene, expression increased after inoculation in both isolates. The CM–CZ difference was not supported at 1 dpi but was supported at 3 and 6 dpi in the RT-qPCR analysis, with higher expression observed in CM. Similarly, the representative A1 aspartic protease-related gene showed no supported CM–CZ difference at 1 dpi but showed higher expression in CM than in CZ at 3 and 6 dpi in this analysis. Together, these RT-qPCR results indicate that most selected colonization-associated candidate genes showed stronger post-inoculation expression in the CM isolate than in the CZ isolate within this experiment, particularly at 3 and 6 dpi. Together, these RT-qPCR results independently corroborate the temporal expression profiles of the selected RNA-seq-derived candidates and prioritize these genes for subsequent functional analysis of maize colonization.

To further evaluate the consistency of the temporal expression patterns obtained by RNA-seq and RT-qPCR, mean RNA-seq FPKM and RT-qPCR relative expression profiles were compared directly for each selected candidate within each isolate (Figure S8). Most candidate–isolate combinations showed broadly similar temporal expression trends between RNA-seq and RT-qPCR. Positive descriptive Pearson correlations between the two assays were observed for all candidate–isolate profiles.

## 4. Discussion

Previous comparative genomic analyses of *C. zeae-maydis* and *C. zeina* identified variation in pathogenicity-associated gene repertoires [7]. In the present study, the public *C. zeae-maydis* SCOH1-5 and *C. zeina* CMW25467 v3 genomes provided the mapping and annotation framework for a matched time-course analysis of pathogen-derived transcriptional responses in two representative Jilin isolates. The combined phenotypic and transcriptomic data revealed both shared and contrasting colonization-associated patterns. Both isolates activated host-associated programs involving carbohydrate and lipid metabolism, redox processes, protease-related functions, CWDEs, secreted effectors, and cercosporin biosynthesis, whereas CM developed visible symptoms earlier and showed stronger or earlier activation of several candidate host-interaction transcripts. These findings identify a common transcriptional framework for maize colonization together with isolate-associated differences in the timing and magnitude of individual candidate responses.

Recent transcriptomic work described *C. zeina* infection as involving a latent or stealth-like phase during maize colonization, characterized by limited early symptom development and stage-specific expression of candidate effectors [42]. In our dataset, CZ showed higher pathogen-derived read mapping rates than CM at 0 and 1 dpi, consistent with the higher inoculum input for CZ, whereas CM showed greater pathogen-derived transcript representation at 6 dpi. Because pathogen-derived read proportions in mixed host–pathogen RNA-seq libraries can be influenced by inoculum deposition, host tissue composition, alignment efficiency, and divergence from the reference genome, they should not be interpreted as direct measurements of fungal biomass. Nevertheless, the later increase in CM-derived transcript representation, together with stronger lesion development, is consistent with faster symptom-associated colonization by CM under the controlled inoculation conditions. Conversely, the lower CZ-derived transcript representation and weaker visible necrosis at 6 dpi are compatible with a more latent-like colonization profile under these conditions.

The controlled inoculation assay reproduced rectangular gray leaf spot lesions delimited by leaf veins following inoculation with both representative isolates. Although RNA-seq sampling ended at 6 dpi, extended observation showed that CM- and CZ-inoculated leaves developed characteristic lesions at 15 and 20 dpi, respectively (Figure S1E,F). Together with multilocus phylogenetic identification and the recovery of pathogen-derived reads from inoculated leaf tissues, these observations support that the transcriptomic profiles were derived from the inoculated *Cercospora* isolates during maize leaf colonization.

A major outcome of this study is that the two Jilin isolates expressed candidate transcripts from several shared host-interaction families, including Ecp6-like, Ecp2-like, Ecp20-2, Nis1-like, NLP/NPP1-like, PsXEG1-like, SsCut1-like, GH5, and GH11 candidates. The LysM effector Ecp6 from *Cladosporium fulvum* suppresses chitin-triggered immunity by sequestering extracellular chitin fragments and attenuating chitin-triggered defense responses [15]. Nis1 family effectors can interfere with plant immune signaling [62], whereas NLP/NPP1-like proteins are widely associated with necrosis induction and immune activation in plant-associated microbes [63]. The post-inoculation expression of PsXEG1-like and SsCut1-like homologs further points to secreted proteins acting at the interface between host cell wall modification and immune interaction [64]. GH5 and GH11 family enzymes contribute to degradation of cellulose- and xylan-containing plant cell wall components and are commonly activated during host colonization by filamentous pathogens [10,11]. RT-qPCR reproduced the temporal expression trends of selected candidates. Together, these results indicate that both isolates deploy overlapping extracellular immune-interaction and cell wall-modification functions during maize colonization, while individual candidates differ between isolates in the timing or magnitude of expression.

Beyond this shared host-interaction toolkit, stronger activation of selected CWDE- and effector-associated transcripts in CM coincided with faster lesion development. In particular, several CM candidates showed earlier or greater post-inoculation expression during the transition from early colonization to visible symptom formation. Similar stage-dependent transcriptional shifts involving hydrolytic enzymes and secreted pathogenicity factors have been reported in other filamentous phytopathogens [11,65,66]. The observed association supports a link between stronger host-associated activation of selected virulence-related candidates and faster symptom development in the CM isolate, although it does not by itself demonstrate a direct causal relationship. The earlier symptom development of CM is also consistent with previous phenotypic observations that *C. zeae-maydis* can grow faster than *C. zeina* under culture conditions [6]. However, in vitro growth rate, in planta colonization dynamics, and visible lesion development represent different biological measurements. The observed contrasts should therefore be interpreted as isolate-associated differences under the controlled inoculation system rather than as fixed species-wide characteristics.

The analysis of genes associated with cercosporin biosynthesis provided additional context for toxin-related transcription during maize colonization. Homologs of the 12 annotated *C. beticola* genes associated with cercosporin biosynthesis were represented in both public reference genomes, and several corresponding transcripts showed different temporal expression patterns between the two Jilin isolates. The shorter CTB7 homolog in the *C. zeina* reference genome has been reported previously and associated with impaired in vitro cercosporin production [24]. The contribution of the present study is the time-resolved comparison of cercosporin biosynthesis-associated transcript expression during maize colonization by the two Jilin isolates. These results indicate isolate-associated differences in the temporal regulation of cercosporin biosynthesis-related genes under the controlled inoculation conditions. Direct determination of CTB7 structure and cercosporin production in the Jilin isolates will require isolate-specific genome sequence and metabolite data.

This study has four principal limitations. First, one isolate per species was analyzed, so the observed contrasts define isolate-associated patterns that require evaluation across broader isolate collections. Second, needleless syringe-assisted inoculation generated synchronized host-associated samples and reproduced characteristic GLS lesions, but bypassed aspects of natural epiphytic development and stomatal entry. Because *Cercospora* conidia are generally larger than stomatal openings, natural entry is more likely to occur through germ tubes or hyphae than through intact conidia. Third, the 0 dpi samples were collected 30 min after inoculation and therefore represent an inoculum-associated baseline rather than an uninoculated or culture-free control. Pathogen-derived transcripts detected at 0 dpi likely originated mainly from deposited conidia and other inoculum-associated fungal material, including material that may have been introduced into substomatal or intercellular spaces during needleless syringe-assisted application. Because the inoculum was produced on maize leaf powder sporulation medium, some transcripts may also reflect the physiological state carried over from in vitro sporulation. Consequently, low baseline counts and inoculum history may influence fold-change estimates. Low-expression filtering, temporal profiles, functional annotation, and RT-qPCR validation were therefore considered together when prioritizing post-inoculation-induced candidates. Because CM and CZ differed in colonization dynamics, identical dpi values represent chronological sampling points rather than strictly matched physiological stages. Fourth, pathogen-mapped read proportions quantify pathogen-derived transcript representation in mixed host–pathogen libraries and are not direct measurements of fungal biomass. Broader isolate collections, additional maize genotypes, complementary inoculation systems, and functional validation will determine which candidate patterns generalize across GLS interactions.

## 5. Conclusions

In summary, this study establishes a reference-guided, pathogen-focused time-course comparison of two representative Jilin *Cercospora* isolates during maize leaf colonization. The CM isolate displayed faster visible symptom development, greater pathogen-derived transcript representation at 6 dpi. Several CWDE- and effector-associated candidates showed stronger or earlier post-inoculation expression in CM. Both isolates activated candidate transcripts related to carbohydrate and lipid metabolism, redox processes, protease-related functions, secretion, and cercosporin biosynthesis, with candidate-specific differences in timing or magnitude. RT-qPCR supported the temporal profiles of selected RNA-seq-derived candidates. Together, these findings connect contrasting colonization behavior with distinct temporal deployment of candidate host-interaction transcripts and provide a prioritized set of genes for functional analysis across expanded isolate collections and independently repeated infection experiments.

## Figures and Tables

**Table 1:** RNA-seq mapping statistics across time points during maize B73 infection by *C. zeae-maydis* and *C. zeina*.

Treatment	Time Point	*n*	No. of Total Reads ^a^	No. of Reads Mapped to B73v5	No. of Reads Mapped to B73v5 (%)	No. of Reads Mapped to Pathogen ^b^	No. of Reads Mapped to Pathogen (%)
*C. zeae-maydis*	0 dpi	3	42,072,121 ± 596,139	41,168,475 ± 578,192	97.85 ± 0.02	13,968 ± 1706	0.04 ± 0.00
*C. zeae-maydis*	1 dpi	3	43,367,121 ± 834,485	42,489,435 ± 803,408	97.98 ± 0.04	16,947 ± 3221	0.04 ± 0.01
*C. zeae-maydis*	3 dpi	3	43,649,485 ± 214,912	42,432,218 ± 164,370	97.21 ± 0.38	106,008 ± 40,471	0.24 ± 0.09
*C. zeae-maydis*	6 dpi	3	45,161,131 ± 1,573,081	43,447,302 ± 1,319,988	96.23 ± 0.42	766,106 ± 214,490	1.67 ± 0.40
*C. zeae-maydis*	in vitro	3	38,172,094 ± 1,703,527	—	—	37,201,101 ± 1,699,303	97.45 ± 0.11
*C. zeina*	0 dpi	3	45,792,330 ± 1,959,382	44,782,423 ± 1,923,628	97.79 ± 0.03	89,762 ± 6744	0.20 ± 0.02
*C. zeina*	1 dpi	3	43,701,703 ± 1,362,132	42,575,838 ± 1,421,967	97.41 ± 0.23	98,032 ± 11,017	0.23 ± 0.02
*C. zeina*	3 dpi	3	47,956,711 ± 2,222,749	46,713,747 ± 2,117,640	97.42 ± 0.14	141,074 ± 9039	0.29 ± 0.01
*C. zeina*	6 dpi	3	44,332,058 ± 524,515	43,176,867 ± 518,723	97.40 ± 0.06	269,666 ± 29,500	0.61 ± 0.06
*C. zeina*	in vitro	3	41,665,339 ± 1,440,430	—	—	40,754,882 ± 1,374,899	97.82 ± 0.13

^a^ Values represent mean ± standard error from three independently processed pooled samples per treatment and time point within a single controlled inoculation experiment. These samples were not derived from independently repeated inoculation experiments and should therefore be interpreted as independently processed pooled samples. ^b^ Pathogen-mapped reads were calculated against the *C. zeae-maydis* SCOH1-5 genome or the *C. zeina* CMW25467 v3 genome.

**Table 2:** Representative reference-annotated candidate secreted proteins, effector homologs, and pathogenicity-related proteins used to assign putative identities to transcripts detected in the two Jilin isolate RNA-seq datasets ^a^.

Orthogroup	Candidate Homolog	Predicted Function	Reference Organism	Identity (%)	E-Value	PHI-Base Phenotype
OG0000299	ECP2-like	Pathogen effector	*Fulvia fulva*	55	5.75 × 10^−52^	reduced virulence effector
OG0002164	Ecp6-like	LysM domain	*Fulvia fulva; Fusarium oxysporum*	48.8	4.51 × 10^−74^	effector
OG0000492	Ecp20-2-like	—	*Fulvia fulva*	42.6	4.98 × 10^−29^	effector
OG0006941	SsCut1 (SS1G 08104)	Cutinase	*Sclerotinia sclerotiorum*	56.4	1.90 × 10^−84^	reduced virulence
OG0008208	Nis1-like	—	*Cercospora beticola* (NCBI BLAST) ^b^	43.1	3.02 × 10^−24^	—
OG0001379	FGSG 08238	—	*Fusarium graminearum*	48.6	7.86 × 10^−38^	unaffected pathogenicity
OG0004782	XYN11A	glycosyl hydrolase 11 family	*Botrytis cinerea*	54.8	1.27 × 10^−52^	reduced virulence
OG0002365	EMP1	Ser-Thr-rich glycosylphosphatidylinositol-anchored membrane family	*Magnaporthe oryzae*	42.5	1.50 × 10^−11^	reduced virulence
OG0000107	BGL2-like	glycosyl hydrolase 17 family	*Candida albicans*	39.3	5.03 × 10^−67^	reduced virulence
OG0001101	PsXEG1-like	glycosyl hydrolase 12 family	*Phytophthora sojae*	53.1	1.04 × 10^−72^	effector
OG0002278	Fpr2 (EJP70756)	FKBP-type peptidyl-prolyl cis-trans isomerase	*Beauveria bassiana*	52	3.80 × 10^−36^	reduced virulence
OG0006905 (CZ-reference-specific)	FlcA-like	—	*Aspergillus fumigatus*	31.1	2.33 × 10^−9^	loss of pathogenicity
OG0001272	XYL2	glycosyl hydrolase 11 (cellulase G) family	*Bipolaris zeicola*	75.5	3.18 × 10^−105^	reduced virulence

^a^ This table summarizes representative reference-annotated candidate secreted proteins, effector homologs, CWDE-associated proteins, and pathogenicity-related proteins used to assign putative identities to expressed transcripts detected in the Jilin isolate RNA-seq datasets. Gene identifiers correspond to reference gene models from *C. zeae-maydis* SCOH1-5 or *C. zeina* CMW25467 v3 to which Jilin isolate-derived reads were mapped. The listed candidates do not constitute confirmed gene models from the Jilin isolate genomes. ^b^ Homology to Nis1-like proteins was identified by NCBI BLAST searches against the non-redundant protein database.

## Data Availability

The raw in vitro and in planta RNA-seq reads generated for this study are available in the NCBI Sequence Read Archive under BioProject accession number PRJNA1463572. The analysis scripts and workflow files used for RNA-seq processing, differential expression analysis, GO enrichment, functional annotation, effector prediction, multilocus phylogenetic analysis, heatmap visualization, and RT-qPCR/RNA-seq correlation analysis are available at GitHub release v1.0.0: https://github.com/lizhaoreng/CM_CZ_RNAseq_analysis_pipeline (accessed on 23 August 2026).

## Supplementary Materials

The following supporting information can be downloaded at: https://www.mdpi.com/article/10.3390/jof12090650/s1, Figure S1: Colony morphology, sporulation culture, and late-stage development of rectangular gray leaf spot lesions by representative *C. zeae-maydis* and *C. zeina* isolates; Figure S2: Multilocus phylogenetic placement and pairwise genetic distances of the two *Cercospora* isolates used in this study; Figure S3: Principal component analysis (PCA) of pathogen-focused RNA-seq samples; Figure S4: Reference-species phylogeny of *Cercospora zeae-maydis* SCOH1-5, *Cercospora zeina* CMW25467 v3, *Cercospora beticola* Cb09-40, *Cercospora kikuchii* MAFF 305040, *Pseudocercospora fijiensis* CIRAD86, and *Zymoseptoria tritici* IPO323; Figure S5: Gene Ontology (GO) enrichment analysis of upregulated reference-annotated transcripts detected in the two Jilin isolate RNA-seq datasets; Figure S6: Reference-based annotation summary of CWDE-associated candidate transcripts detected in the two Jilin isolate RNA-seq datasets; Figure S7: Heatmap of reference-guided expression patterns for representative candidate effector-associated transcripts detected in the two Jilin isolate RNA-seq datasets; Figure S8: Time-course comparison of RNA-seq and RT-qPCR expression profiles for selected colonization-associated candidate transcripts; Table S1: Accession numbers and sources of marker sequences used for multilocus phylogenetic analysis; Table S2: Reference-based orthogroup classification, member proteins, and copy numbers in the public *C. zeae-maydis* SCOH1-5 and *C. zeina* CMW25467 v3 reference annotations; Table S3: Exploratory DESeq2 differential-expression results, raw-count-based statistics, and functional annotations for each post-inoculation time point relative to 0 dpi in the representative *C. zeae-maydis* and *C. zeina* Jilin isolate datasets; Table S4: Gene Ontology enrichment analysis of reference-annotated transcripts meeting the exploratory upregulation criteria during maize leaf colonization in the two Jilin isolate datasets; Table S5: Reference-based orthogroup assignments and CAZy annotations of putative cell wall-degrading enzyme (CWDE)-associated candidates, together with mapped-transcript expression profiles in the representative *C. zeae-maydis* and *C. zeina* Jilin isolate datasets; Table S6: Reference-based orthogroup assignments, effector predictions, and functional annotations of candidate effector-associated loci, together with mapped-transcript expression profiles in the representative *C. zeae-maydis* and *C. zeina* Jilin isolate datasets; Table S7: Homology-based identification of reference-annotated genes associated with cercosporin biosynthesis, together with mapped-transcript expression profiles in the representative *C. zeae-maydis* and *C. zeina* Jilin isolate datasets; Table S8: Reference-guided functional annotations and mapped-transcript expression profiles of candidate transcripts associated with fatty acid β-oxidation, the glyoxylate cycle, and related lipid metabolic processes in the representative *C. zeae-maydis* and *C. zeina* Jilin isolate datasets; Table S9: Reference-based orthogroup assignments and MEROPS-based annotations of protease and protease-related candidates, together with mapped-transcript expression profiles in the representative *C. zeae-maydis* and *C. zeina* Jilin isolate datasets; Table S10: Primers used for RT-qPCR validation of selected candidate genes.
