# Supplementary material for: Time-Course Comparative Transcriptomics Between Isolates of Cercospora zeae-maydis and Cercospora zeina During Maize Infection
Source: J Fungi (Basel). 2026 Sep 1;12(9):650. doi: 10.3390/jof12090650 (PMC13608403; doi:10.3390/jof12090650)
Supplement: Supplementary file 1 [file jof-12-00650-s001.zip › Table S10.pdf]

Table S10. Primers used for RT-qPCR validation of selected candidate genes.

Reason for primer	Primer identifier	Protein Identifier	Sequence (5' – 3')	Amplicon size (bp)
GH11	CM_GH11_F	KAF2208979	AGGAGCGTGCCACTCTCAA	318
GH11	CM_GH11_R		CATCCGAAGTGACCGTGCC	318
GH11	CZ_GH11_F	KAM3424045.1	AATGGTGCTGCTGCGAAC	267
GH11	CZ_GH11_R		GCCTTGTTGCGTGACTCCA	267
GH5	CM_GH5_F	KAF2214109	TGGCGACTACCTGCGACAG	192
GH5	CM_GH5_R		GGCGTCTCCGTCAACTCATA	192
GH5	CZ_GH5_F	KAM3416242.1	ACCTGTTGGCTGGCAGTTC	186
GH5	CZ_GH5_R		GCATTGGTCGGTCCACCTT	186
Ecp6-like	CM_Ecp6-like_F	KAF2212521	AACCAACCGTCAGGAGGCT	240
Ecp6-like	CM_Ecp6-like_R		GGTGAGGCAAGAGGTGTTGT	240
Ecp6-like	CZ_Ecp6-like_F	KAM3415019.2	GCCAGCAACGTCCAGAATGT	189
Ecp6-like	CZ_Ecp6-like_R		AGCGATGGAGGTGAAGGTGT	189
Nis1-like	CM_Nis1-like_F	KAF2217041	TCGGCTTCTACGCACCCAA	291
Nis1-like	CM_Nis1-like_R		TCCGCTCGGCACCAATAAGA	291
Nis1-like	CZ_Nis1-like_F	KAM3424505.1	TCGTCGGCTTCTACGCACC	312
Nis1-like	CZ_Nis1-like_R		ACACCTGACTCAACGGGCT	312
NPP1-like	CM_NPP1_F	KAF2209457	GGCTCTTCAGATGGCTGCT	327
NPP1-like	CM_NPP1_R		TCCGCCTTGAGTGCTCGTG	327
NPP1-like	CZ_NPP1_F	KAM3421146.1	CGTCAACTGGACTCGGACAT	267
NPP1-like	CZ_NPP1_R		CCGTCTTCGCAGCAGGATT	267
Reference	CM_β-tubulin_F	KAF2214652	ACCACCTTGTCTCTGCCGT	270
Reference	CM_β-tubulin_R		TAGCCGAGCAAGTGAGGTAG	270

Reference	CZ_β-tubulin_F	KAM3418835.1	TGAGGCTGAAGGCTGCGAC	154
Reference	CZ_β-tubulin_R		TGGCGATGGCATGACGGAG	154

Primers were designed for RT-qPCR validation of selected candidate genes associated with cell wall-degrading enzymes, candidate effectors, and reference-gene normalization. CM and CZ indicate the representative *C. zeae-maydis* and *C. zeina* isolates, respectively. β-tubulin was used as the internal reference gene for normalization. Amplicon sizes were determined based on the corresponding target sequences.
